# Clinical outcomes of endovascular aneurysm repair of abdominal aortic aneurysm complicated with hypertension: A 5-year experience

**DOI:** 10.12669/pjms.321.7966

**Published:** 2016

**Authors:** Xi-Tao Peng, Qi-Dong Yuan, Ming-Zhe Cui, Hong-Chao Fang

**Affiliations:** 1 Xi-Tao Peng, He’nan Provincial People’s Third Hospital, Zhengzhou 450006, P. R. China; 2 Qi-Dong Yuan, He’nan Provincial People’s Third Hospital, Zhengzhou 450006, P. R. China; 3 Ming-Zhe Cui, He’nan Provincial People’s Third Hospital, Zhengzhou 450006, P. R. China; 4 Hong-Chao Fang, He’nan Provincial People’s Third Hospital, Zhengzhou 450006, P. R. China

**Keywords:** Endovascular aneurysm repair, Abdominal aortic aneurysm, Hypertension, artificial blood vessel replacement, Quality of life

## Abstract

**Objective:**

To evaluate the therapeutic effects of endovascular aneurysm repair (EVAR) on abdominal aortic aneurysm (AAA) complicated with hypertension.

**Methods:**

Fifty-two patients with AAA complicated with hypertension treated in our hospital were retrospectively analyzed. They were divided into an observation group (34 cases) and a control group (18 cases). The control group was treated by incision of AAA and artificial blood vessel replacement, and the observation group was treated by EVAR.

**Results:**

All surgeries were performed successfully. However, compared with the control group, the observation group had significantly less surgical time, intraoperative blood loss and blood transfusion, as well as significantly higher total hospitalization expense (P<0.05). During the one-month follow-up, the observation group was significantly less prone to pulmonary infection, surgical site infection, lower-extremity deep venous thrombosis and lower extremity weakness than the control group (P<0.05). The observation group enjoyed significantly better quality of life than the control group did one and three months after surgery (P<0.05).

**Conclusion:**

Given sufficient funding, EVAR should be preferentially selected in the treatment of AAA complicated with hypertension due to minimal invasion, safety, stable postoperative vital signs and improved quality of life.

## INTRODUCTION

Aortic aneurysm, although infrequent in clinical practice [e.g. incidence of primary abdominal aortic aneurysm (AAA): only 2%], leads to poor prognosis and high mortality rate.^1^ In general, aneurysm locally dilates normal artery by over 50% in the diameter. Particularly, the abdominal aorta diametered >3 cm is referred to as AAA.^2^,^3^ On the other hand, hypertension is the main risk factor for AAA, especially in the patients aged about 65 years old. AAA complicated with hypertension, when ruptured, gives rise to extremely high mortality rate (i.e. over 90%), thus requiring surgical treatment based on proper indications.^4^

Since the 1990s, patients have been treated with minimally invasive surgeries as the standard method which, however, results in traumas, severe postoperative cardiac and pulmonary complications or slow recovery. With the development of surgical techniques, anesthetic monitoring and perioperative care, endovascular aneurysm repair (EVAR) has been widely pereformed in clinical practice and it excels open surgeries in lowering the mortality and morbidity rates.^5^-^7^ It is now well-accepted that traditional surgery is suitable for the hypertension patients with infrarenal AAA. However, EVAR is restricted by anatomical characteristics and plaques of the aneurysm neck, angulation of the iliac artery, degree of calcification, and blood supply of important arterial branches.^8^ Hence, we herein analyzed the therapeutic effects of EVAR on AAA complicated with hypertension.

## METHODS

### Subjects

Fifty-two patients with AAA complicated with hypertension treated in our hospital from February 2008 to January 2013 were selected. This study was approved by the ethics committee of He’nan Provincial People’s Third Hospital. Written consent has been obtained from all patients.

### Inclusion criteria

Patients conforming to the diagnostic standards for AAA complicated with hypertension; patients with touchable and painless pulsating masses in the abdomen; patients with infrarenal AAA sized lower than 5 cm and requiring treatment.

### Exclusion criteria

Patients complicated with severe hepatic and renal diseases; patients complicated with mental diseases; pregnant women; patients younger than 20 years old.

The patients were divided into an observation group (34 cases) and a control group (18 cases). The two groups had similar gender ratio, age, aneurysm size, number of patients who smoked or drank, systolic blood pressure and diastolic blood pressure (P>0.05) (Table-I).

**Table-I:** Basic clinical data of the two groups.

Index	Observation group (n=34)	Control group (n=18)	χ^2^ or t	P
Gender (male/female)	32/28	31/29	0.064	>0.05
Age (years old)	63.23±2.89	63.19±3.19	0.078	>0.05
Aneurysm size (cm)	5.56±1.09	5.58±1.11	0.034	>0.05
Smoking patients	28 (46.7%)	29 (48.3%)	0.043	>0.05
Drinking patients	21 (35.0%)	20 (33.3%)	0.067	>0.05
Systolic pressure (mmHg)	159.33±11.98	160.09±12.78	0.119	>0.05
Diastolic pressure (mmHg)	97.19±9.23	97.56±8.91	0.098	>0.05

### Surgical Methods

Control group: The patients were treated by incision of AAA and artificial blood vessel replacement. Under general anesthesia, soft tissues were separated layer-by-layer after laparotomy, and the posterior peritoneum was cut open to expose AAA. Then the proximal- and distant-end aneurysm necks were blocked, and the aneurysm anterior wall was cut open to ligate lumbar artery and inferior mesenteric artery openings. Afterwards, appropriate artificial blood vessels were transplanted, and blood flow was recovered after inosculation. In the case these artificial blood vessels were wrapped by AAA, the abdominal incision was closed.

### Observation group

The observation group was treated by EVAR. Under local anesthesia, the patients received EVAR in an operating room equipped with angiography devices. Approximately 5 cm long oblique incisions were made in bilateral inguinal regions to expose bilateral femoral arteries. Angiography for the abdominal aorta was conducted with the Seldinger technique to determine whether EVAR should be performed. Subsequently, a proper covered stent was selected and located at an appropriate position of the abdominal aorta under fluoroscopy. Thereafter the covered stent was released to make the anchor region adhere tightly to the wall, during which the blood flow outcomes were observed by angiography. Finally, the incisions were repaired.

### Observation Indices

Perioperative observation: The surgical time, intraoperative blood loss and blood transfusion and total hospitalization expense of the two groups were observed. Criteria for successful EVAR: AAA was isolated without ruptures, and blood flowed smoothly inside the covered stent. Criteria for successful incision of AAA and artificial blood vessel replacement: Bloods flowed smoothly inside both the abdominal aorta and artificial blood vessels, without the latters infected.

### Complications

Complications such as pulmonary infection, surgical site infection, lower-extremity deep venous thrombosis and lower extremity weakness were observed in the postoperative 1st month.

### Quality of life

The quality of life was investigated by SF-36 scale in the postoperative 1st and 3rd months, and a higher total score means better quality of life.

### Statistical Analysis

All data were analyzed by SPSS 15.0. The categorical data were expressed as (*x*±*s*), and inter-group comparisons were performed by independent samples t-test. The numerical data were expressed as case numbers or composition ratios and compared by Chi-square test. P<0.05 was considered statistically significant.

## RESULTS

### Perioperative Indices

Compared with the control group, the observation group had significantly less surgical time, intraoperative blood loss and blood transfusion, as well as significantly higher total hospitalization expense (P<0.05) (Table-II).

**Table-II:** Perioperative indices (*x*±*s*).

Index	Observation group (n=34)	Control group (n=18)	t	P
Surgical time (min)	146.34±10.34	210.98±15.39	6.988	<0.05
Intraoperative blood loss (ml)	50.56±11.98	1000.89±150.32	12.983	<0.05
Blood transfusion (ml)	100.98±12.11	800.18±187.18	8.397	<0.05
Total hospitalization expense (CNY)	138023±1593.98	38453.20±1932.78	11.867	<0.05

### Postoperative Complications

During the one-month follow-up, the observation group was significantly less prone to pulmonary infection, surgical site infection, lower-extremity deep venous thrombosis and lower extremity weakness than the control group (P<0.05) (Table-III).

**Table-III:** Postoperative complications (n).

Index	Observation group (n=34)	Control group (n=18)	χ^2^	P
Pulmonary infection	0	3		
Surgical site infection	1	4		
Lower-extremity deep venous thrombosis	0	3		
Lower extremity weakness	1	3		

Total	2 (3.3%)	12 (20.0%)	7.113	<0.05

### Quality of Life

The observation group enjoyed significantly better quality of life than the control group one and three months after surgery (P<0.05) (Table-IV).

**Table-IV:** Postoperative quality of life.

Index	Observation group (n=34)	Control group (n=18)	t	P
Postoperative 1st month	80.98±3.71	67.82±4.11	12.081	<0.05
Postoperative 3rd month	88.89±4.21	73.29±4.98	13.992	<0.05

### Case Analysis

**Fig.1:**
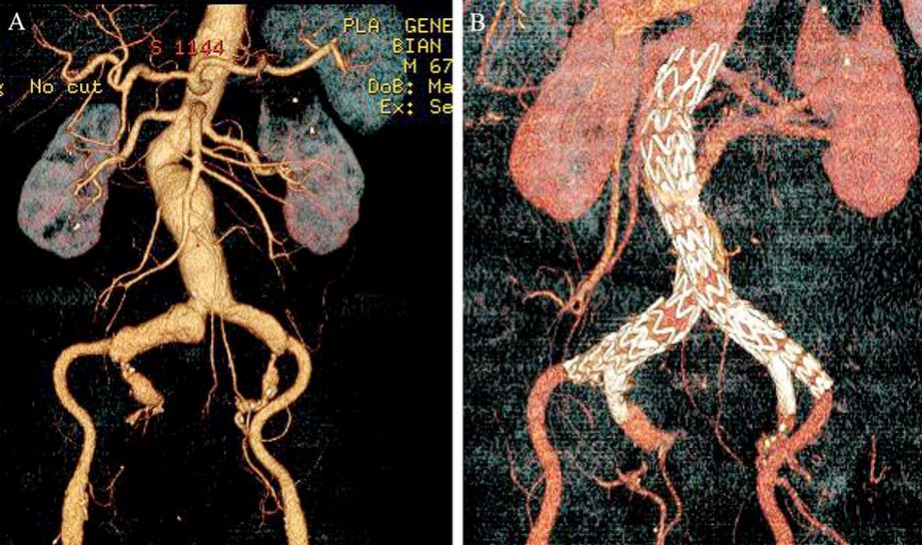
CT images for one patient who received EVAR. A: Preoperative CT discloses AAA complicated with bilateral iliac aneurysms; B: Re-examination in the postoperative 6th month shows well-maintained stent form without endoleak, and unobstructed bilateral internal iliac arteries.

**Fig.2:**
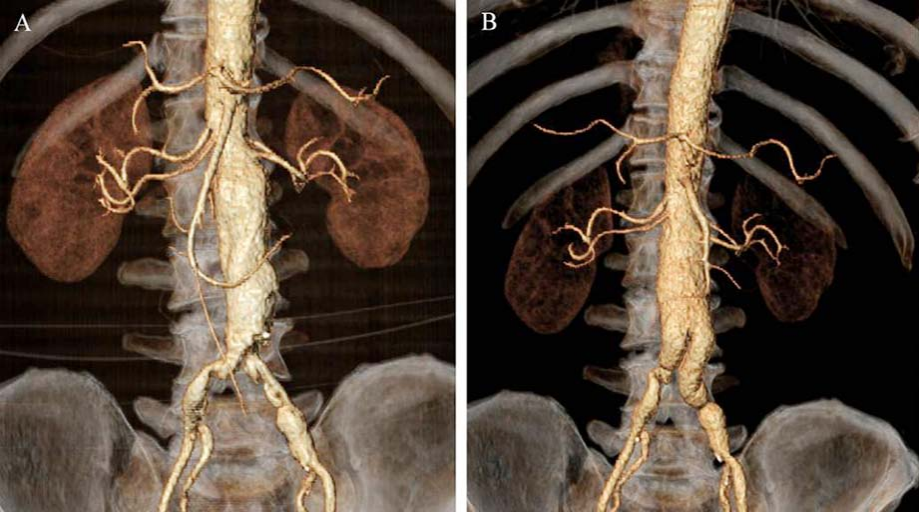
CT images for one patient who received traditional surgery. A: Preoperative CT discloses AAA; B: Re-examination in the postoperative 6th month shows unobstructed artificial blood vessels, proximal-end abdominal aorta anastomotic stoma and distal-end common iliac artery anastomotic stoma.

CT images for the patients who received EVAR and traditional surgery are shown in Fig. 1 and 2 respectively.

## DISCUSSION

AAA complicated with hypertension, which is a severe aortic incident threatening human life, should be treated as early as possible to prevent rupture of the bulge. Despite improved surgical and anesthetic techniques, this disease is challenging for clinicians worldwide.^9^

Incision of AAA in combination with artificial blood vessel replacement majorly traumatic and may lead to a variety of complications.^10^ Contrarily, EVAR is performed under real-time monitoring to lead an appropriate covered stent into the abdominal aorta through the femoral artery, aiming to cover the proximal- and distal-end aneurysm necks. As a result, the wall of AAA is isolated from blood flow inside vascular lumen, whereas blood flowed smoothly inside the covered stent, thus maintaining smooth blood flow in the abdominal aorta.^11^ The observation group had significantly less surgical time, intraoperative blood loss and blood transfusion, as well as significantly higher total hospitalization expense compared with the control group did (P<0.05). The results suggested that EVAR obviously decreased the intraoperative blood loss and blood transfusion, and considerably shortened the hospitalization stay. In other words, this method barely interfered with the circulatory system or led to traumas, and managed to reduce the risks of blood transfusion complications. Meanwhile, the observation group had more stable vital signs and recovered more rapidly. Nevertheless, EVAR is limited in clinical practice due to high expenses.^12^,^13^

During one month of follow-up, the observation group suffered from significantly less complications such as pulmonary infection, surgical site infection, lower-extremity deep venous thrombosis and lower extremity weakness than the control group did (P<0.05). Probably, the observation group was not endangered by complications owing to minor traumas and short hospitalization stay. Notably, the surgical time, especially that for artery occlusion, should be minimized to decrease the risks of infection. Aortic CT angiography or magnetic resonance angiography should be performed to target aortic lesions, and to provide reference for the design of treatment protocols and the selection of stent diameter and length.^14^ It is important to lower blood pressure before releasing the stent to prevent its displacement upon the impact of high-speed blood flow. When released, a covered stent, which has better compliance at the distal end than that at the proximal end as well as larger distal-end diameter than proximal-end one, is highly recommended.^15^

SF-36 scale, also known as short-form health survey, is designed to investigate the quality of life and to evaluate the treatment outcomes by analyzing the postoperative psychological and physical health states, thus having been widely applied to assess the health status of target patients.^16^,^17^ The observation group showed significantly better quality of life than the control group did one and three months after surgery (P<0.05).

In summary, with sufficient funding, EVAR can effectively treat AAA complicated with hypertension minimally invasively, giving rise to stable postoperative vital signs and improving the quality of life.
